# Investigation of the association between *CALCRL* polymorphisms and primary angle closure glaucoma

**Published:** 2009-10-27

**Authors:** Dan Cao, Xing Liu, Xiangming Guo, Yanhong Cong, Jingjing Huang, Zhen Mao

**Affiliations:** State Key Laboratory of Ophthalmology, Zhongshan Ophthalmic Center, Sun Yat-Sen University, Guangzhou, People’s Republic of China

## Abstract

**Purpose:**

To determine whether the polymorphisms of calcitonin receptor-like receptor gene (*CALCRL*) are associated with primary angle closure glaucoma (PACG) in a southern Chinese population.

**Methods:**

A total of 207 individuals with acute and chronic PACG and 205 ethnically matched controls were recruited in the current study. A tag-single nucleotide polymorphism approach was used to investigate this gene. Alleles were determined by PCR restriction fragment length polymorphism.

**Results:**

The results show a nominal association between rs1157699 and acute PACG (uncorrected p=0.024 and 0.028 for alleles and genotypes, respectively). Haplotype T_rs840617_C_rs6759535_T_rs1157699_ frequency is significantly higher in acute PACG patients than in controls (corrected p=0.012), whereas haplotype T_rs840617_C_rs6759535_C_rs1157699_ frequency is significantly lower in acute PACG patients compared with controls (corrected p=0.02). However, no significant difference was detected between chronic PACG and *CALCRL* tag single nucleotide polymorphisms.

**Conclusions:**

The study suggests a possible role of *CALCRL* in the pathogenesis of acute PACG but not chronic PACG. A replication of this study with a larger sample size as well as on different populations will be helpful in confirming this finding.

## Introduction

Glaucoma is the second leading cause of blindness worldwide, and 60 million people will have open angle glaucoma and angle closure glaucoma (ACG) by 2010 [1]. Primary angle closure glaucoma (PACG) is the most common type of glaucoma in the Asian population and is responsible for the vast majority of bilateral glaucoma blindness in China [2]. PACG can be divided into an acute or chronic form. Acute PACG (APACG) has a rapid onset, dramatic symptoms, and severe elevation of intraocular pressure (IOP), while chronic PACG (CPACG) is latent—the presence of peripheral anterior synechiae is gradually formed, resulting in visual field defects. Although their manifestations are different, APACG and CPACG share similar anatomical characteristics and both are caused by factors that either pull or push the iris up into the angle, physically blocking the drainage of aqueous humor and raising IOP [3].

Predisposing factors for PACG are mainly related to the geometry of the anterior chamber, such as a shallow anterior chamber, narrow angle, lens position, lens thickness, and short axial length [4]. Other risk factors include age, female sex, and family history [5]. Lowe [6] suggested that the action of a large number of grouped or independently inherited genes results in anterior chamber shallowness. However only 10% of people with anatomically narrow angles develop PACG [7]. Therefore other factors may alter the degree of pupillary block, thus triggering a PACG attack. Because the pathogenesis of PACG is complex, there has been no single or combination of parameters that is sensitive enough to predict whether eyes with a shallow anterior chamber and “occludable angle” will develop PACG.

Perhaps, it is reasonable to expect that genes involving development and functioning of the anterior segment participate in the geometry of the anterior chamber. A number of lines of evidence have suggested that both genetic and environmental factors contribute to the development of PACG [6]. PACG, as a common complex disease [8], has become an important target for association studies in recent years. PACG is associated with genes related to regulation of axial length and structural remodeling of connective tissues, such as membrane-type frizzled-related protein (*MFRP*) gene, matrix metalloproteinase 9 (*MMP-9*) gene, and methylenetetrahydrofolate reductase (*MTHFR*) gene [9-13]. The biological significance of the associated single nucleotide polymorphisms (SNPs) in the development of PACG is still unknown [9]. However such association has not been replicated in subsequent research [10,11].

Recently, a transgenic mouse model for acute ACG was generated by Lars et al. [14]. Overexpression of calcitonin receptor-like receptor (*CALCR*L) in the pupillary sphincter muscle resulted in pupillary palsy and acutely and transiently elevated IOP in mice, mimicking the characteristic phenotype of acute PACG in humans. It is unknown whether genetic variations in *CALCRL* are associated with PACG.

In this study, genetic association analysis was performed between tag SNPs of *CALCRL* and PACG (both APACG and CPACG) in a southern Chinese population.

## Methods

### Patient recruitment and assessment

Unrelated PACG patients and control subjects were recruited from the Zhongshan Ophthalmic Center of Sun Yat-sen University (Guangzhou, China). The study subjects consisted of 207 patients with PACG (including 139 females and 68 males , age 50-80) and 205 unaffected controls (including 138 females and 67 males , age 43-95). Written informed consent was obtained from all subjects, and the study had the approval of the Ethics Committees of the Zhongshan Ophthalmic Center and was performed according to the tenets of the Declaration of Helsinki. All control subjects were matched by age and gender with the patients. All study subjects were Han Chinese from southern China.

PACG was defined according to the International Society of Geographical and Epidemiologic Ophthalmology classification by Foster et al. [15]. PACG subjects were further categorized into two groups: APACG and CPACG.

The diagnosis of APACG was based on the following criteria:

(1) the presence of at least two symptoms: eye pain, headache, blurred vision, and vomiting;

(2) the presence of at least three of the following signs: conjunctival congestion, corneal epithelial edema, mid-dilated unreactive pupil, glaucomflecken, and iris atrophy;

(3) 270° or greater of anterior chamber angle closure on gonioscopic examination;

(4) IOP >40 mmHg by Goldmann applanation tonometry.

The diagnosis of CPACG was based on the following criteria:

(1) the presence of glaucomatous optic neuropathy, which was defined as a cup-to-disc ratio ≥0.7 or asymmetry ≥0.2 between the two eyes, neuroretinal rim width reduced to ≤0.1 cup-to-disc ratio, and nerve fiber layer defect;

(2) visual field loss detected with static automated white-on-white threshold perimetry (SITA fast strategy, program 30–2, model 750; Humphrey Field Analyzer; Carl Zeiss Meditec, Dublin, CA) that is consistent with glaucomatous optic nerve damage. This was defined as Glaucoma Hemifield test results outside normal limits and/or an abnormal pattern standard deviation (SD) with p<5% occurrence in the normal population;

(3) a closed angle on indentation gonioscopy. A closed angle was defined as the presence of at least a 180° angle in which the posterior pigmented trabecular meshwork (TM) was not visible on gonioscopy and with evidence of peripheral anterior synechiae in any part of the angle.

Patients were excluded if they had secondary angle closure or a personal history of hypertension, diabetes, or cardiovascular disease.

Control subjects did not have a history of any of the above symptoms and signs. All the control subjects received a complete ocular examination, which included best corrected visual acuity measurements using the logarithm of the minimum angle of resolution 4-m charts, slit-lamp evaluation of the anterior segment, fundus examination, measurement of IOP, axial length measurement (A-scan ultrasonography, Quantel Medical, Clermont-Ferrand, France), and detailed recording of the health and degree of cupping of the optic nerve head. All control subjects had open angles, IOPs of <21 mmHg, normal optic nerve heads with cup-to-disc ratio of ≤0.5, no family history of glaucoma, no ophthalmic diseases except cataract, and no personal history of hypertension, diabetes, or cardiovascular diseases.

### DNA Preparation

DNA samples of PACG patients and controls were prepared from leukocytes of  peripheral venous blood using the phenol-chloroform extracted method and stored at –80 °C until use.

### Tag Single Nucleotide Polymorphism Selection and Genotyping

The Tagger program in Haploview 4.1 was used for selecting tag SNPs. In order to cover most of the genetic variability of the *CALCRL* gene, tag SNPs were chosen using pair-wise tagging, Hardy–Weinberg equilibrium p value cutoff 0.05, r^2^ cutoff >0.8, minimum minor allele frequency >0.1, and population CHB (Han Chinese in Beijing, China)+JPT (Japanese in Tokyo, Japan). Finally we chose seven tag SNPs suggested by the Tagger program, which captured 87.7% of common *CALCRL* SNPs in the Hapmap database. These proposed tag SNPs were: rs7591567 [C/T] at intron 1, rs3821181 [C/G] at intron 1, rs9288141 [A/G] at intron 1, rs1157699 [C/T] at intron 1, rs840617 [A/T] at intron 8, rs6759535 [C/T] at intron 8, and rs3771073 [C/G] at intron 1. The latter two tag SNPs were used in exchange for rs3771081 (D'=1, r^2^=1.0) and rs12104503 (D'=1, r^2^=0.905), respectively.

All seven tag SNPs were genotyped by PCR restriction fragment length polymorphism (PCR-RFLP). The primers of the seven sites were designed using Primer Premier 5.0 software (Premier Biosoft International, Palo Alto, CA). The details of the primers and enzymes used for PCR-RFLP genotyping are presented in Table 1.

**Table 1:** Primer pairs and enzymes used for *CALCRL* tag snp genotyping.

**dbSNP ID**	**Forward primer**	**Reverse primer**	**Product length (bp)**	**Tm (°C)**	**Restriction enzyme**
rs7591567	GTCACGCTGAGGTAGG	GATTTCATTTGCCACC	485	56	BstZ171
rs3771073	CAGAATGTAGCAGGAC	CTCTAAAGGGATGGTT	215	51	Ddel
rs3821181	GATACCTAGGGAACCA	ACAACAGCGAAACATT	280	51	AflII
rs9288141	CCTGCAAGACAATCCC	ATCCGACAAGGTGAGC	381	56.6	HpaII
rs1157699	GATGAAAGCCTGAGAA	ACCTGCCTCCATACTC	165	52.4	BanII
rs6759535	TTTATTGAGTGCCTAC	AAATGGACCATGTTTA	381	50.3	HpaII
rs840617	TCCATTGGCTAAGTCC	CCCTTACCCTTTCCAG	353	54.3	HpyCH4IV

The PCR reaction was performed in a thermocycler (Biometra, GoÈttingen, Germany) under the following conditions: an initial denaturation at 95 °C for 5 min followed by 33 to 35 cycles of 95 °C for 30 s, proper annealing temperature for 30 s, and 72 °C for 30 s. The final extension cycle of 72 °C was for 5 min. The PCR products were then digested by the proper restriction enzymes (New England Biolabs, Inc., Beijing, China). Digestion products were loaded onto an 8% (49:1) polyacrylamide/0.5× Tris/Borate/EDTA gel and resolved at 35 W for about 1.5 h. The images were recorded digitally.

### Statistical Analysis

Statistical analysis was performed with SPSS version 12.0 for Windows (SPSS Inc., Chicago, IL). The Hardy–Weinberg equilibrium was tested by the χ^2^ test. We evaluated the frequency of genotypes and alleles in this study using the χ^2^ test or Fisher’s exact test (p values <0.05 were considered significant). The haplotype frequency and linkage disequilibrium of the SNPs were estimated with Haploview 4.1. A haplotype frequency <0.03 was not studied further. All the data were corrected by Bonferroni correction.

## Results

**Figure 1:**
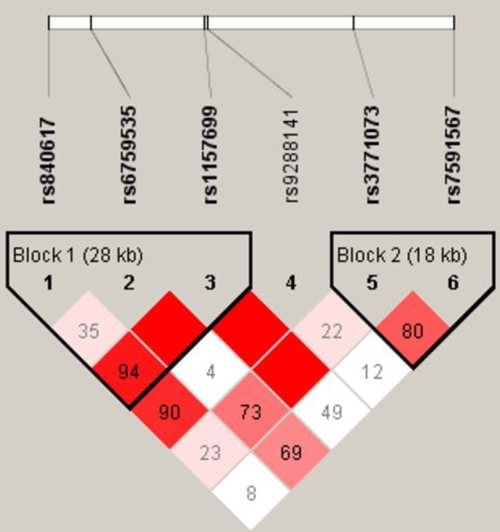
The haplotype block structure and the position of the studied tag single nucleotide polymorphisms of *CALCRL*. The relative physical position of each tag single nucleotide polymorphism (SNP) is given together with the rs number in the upper part of the diagram. Pair-wise SNP D’ values (multiply by 100) of linkage are shown together with two identified haplotype blocks. The bright red rectangles represent D’=100 (multiply by 100).

There was no difference between the control group and the patients with PACG in gender and age, as shown in Table 2. Seven tag SNPs in *CALCRL* were determined in patients and controls. The polymorphism at rs3821181 was not detected in all 412 subjects (C/C genotype in all subjects), so it was excluded in the following comparisons. The other six SNPs were in Hardy–Weinberg equilibrium in APACG and CPACG, as well as in controls (p>0.05). The haplotype block structure, together with D’ values and the position of the studied tag SNPs of *CALCRL*, are shown in Figure 1.

**Table 2:** Demographic features of PACG and control subjects.

**Demograhphic features**		**Control subjects (n=205)**	**APACG subjects (n=109)**	**p value**	**CPACG subjects (n=98)**	**p value**
Gender	Female	138 (67%)	75 (68.8%)	0.788	64 (65.3%)	0.728
	Male	67 (33%)	34 (31.2%)		34 (34.7%)	
Age	Mean±SD	67.12±7.89	65.91±8.66	0.654	65.59±7.16	0.677
	Range	50-80	45-95		43-85	

### Single marker analysis

Allele and genotype frequency comparisons between APACG patients and controls demonstrated nominally significant differences with p values of 0.024 and 0.028, respectively, for the C/T polymorphism at intron 1 (rs1157699), but this significance was lost after Bonferroni correction (corrected p value 0.144 and 0.168, respectively). No significant difference in the remaining five SNPs tested was observed between APACG patients and controls in the distribution of alleles and genotypes (Table 3 and Table 4), and none of the six SNPs were significantly associated with CPACG (Table 3 and Table 4).

**Table 3:** Frequency of genotypes of six *CALCRL* snps in PACG patients and controls.

**dbSNP ID**	**Genotype**	**Control n (%)**	**APACG n (%)**	**p Value**	**CPACG n (%)**	**p Value**
rs7591567	CC	5 (2.4%)	3 (2.8%)	0.779	2 (2%)	0.368
	CT	53 (25.9%)	32 (29.4%)		33 (33.7%)	
	TT	147 (71.7%)	74 (67.9)		63 (64.3%)	
rs3771073	CC	4 (2%)	2 (1.8%)	0.343	1 (1%)	0.509
	CG	57 (27.8%)	39 (35.8%)		33 (33.7%)	
	GG	144 (70.2%)	68 (62.4%)		64 (65.3%)	
rs9288141	AA	128 (62.4%)	68 (62.4%)	0.816	61 (62.2%)	0.951
	AG	72 (35.1%)	37 (33.9%)		34 (34.7%)	
	GG	5 (2.4%)	4 (3.7%)		3 (3.1%)	
rs1157699	CC	138 (67.3%)	57 (52.3%)	0.028	60 (61.2%)	0.415
	CT	60 (29.3%)	48 (44%)		32 (32.7%)	
	TT	7 (3.4%)	4 (3.7%)		6 (6.1%)	
rs6759535	CC	95 (46.3%)	56 (51.4%)	0.469	50 (51%)	0.742
	CT	97 (47.3%)	44 (40.4%)		42 (42.9%)	
	TT	13 (6.3%)	9 (8.3%)		6 (6.1%)	
rs840617	AA	18 (8.8%)	12 (11%)	0.494	5 (5.1%)	0.488
	AT	79 (38.5%)	35 (32.1%)		37 (37.8%)	
	TT	108 (52.7%)	62 (56.9%)		56 (57.1%)	

**Table 4:** Frequency of alleles of six *CALCRL* snps in PACG patients and controls.

**dbSNP ID**	**Allele**	**Control n (%)**	**APACG n (%)**	**p value**
APACG, n(%)				
rs7591567	C	63 (15.4%)	38 (17.4%)	0.502
	T	347 (84.6%)	180 (82.6%)	
rs3771073	C	65 (15.9%)	43 (19.7%)	0.221
	G	345 (84.1%)	175 (80.3%)	
rs9288141	A	328 (80%)	173 (79.4%)	0.849
	G	82 (20%)	45 (20.6%)	
rs1157699	C	336 (82%)	162 (75.2%)	0.024
	T	74 (18%)	56 (24.8%)	
rs6759535	C	287 (70%)	156 (71.6%)	0.683
	T	123 (30%)	62 (28.4%)	
rs840617	A	115 (28%)	59 (27.1%)	0.792
	T	295 (72%)	159 (72.9%)	
CPACG, n(%)				
rs7591567	C	63 (15.4%)	37 (18.9%)	0.276
	T	347 (84.6%)	159 (81.1%)	
rs3771073	C	65 (15.9%)	35 (17.9%)	0.534
	G	345 (84.1%)	161 (82.1%)	
rs9288141	A	328 (80%)	156 (79.6%)	0.910
	G	82 (20%)	40 (20.4%)	
rs1157699	C	336 (82%)	152 (77.6%)	0.201
	T	74 (18%)	44 (22.4%)	
rs6759535	C	287 (70%)	142 (72.4%)	0.535
	T	123 (30%)	54 (27.6%)	
rs840617	A	115 (28%)	47 (24%)	0.289
	T	295 (72%)	149 (76%)	

### Haplotype analysis

Pair-wise linkage disequilibrium between the six tag SNPs is shown in Figure 1. We identified two haplotype blocks. In block 1 the frequency of the T_rs840617_C_rs6759535_T_rs1157699_ haplotype was significantly higher in APACG patients than controls (18.2% versus 9.0%, respectively p=0.0015, Bonferroni corrected p=0.012; Table 5), and the frequency of haplotype T_rs840617_C_rs6759535_C_rs1157699_ was significantly lower in APACG patients compared with controls (31.9% versus 42.4% respectively, p=0.0028, Bonferroni corrected p=0.02; Table 5). No association between other haplotypes and CPACG was found (data not shown).

**Table 5:** Haplotype frequencies in APACG patients and controls.

**Haplotype block**	**Haplotype**	**APACG (frequence)**	**Control (frequence)**	**Fisher’s p**	**Corrected p**	**Odds ratio (95% CI)**
block 1	ACC	35.04 (0.161)	59.35 (0.145)	0.7395	NS	1.08 (0.69-1.70)
	ACT	11.79 (0.054)	16.71 (0.041)	0.5117	NS	1.29 (0.60-2.77)
	ATC	9.73 (0.045)	26.85 (0.065)	0.2398	NS	0.64 (0.30-1.36)
	TCC	69.44 (0.319)	174.03 (0.424)	0.0028	0.02	0.59 (0.42-0.83)
	TCT	39.74 (0.182)	36.92 (0.090)	0.0015	0.012	2.16 (1.33-3.50)
	TTC	49.80 (0.228)	75.78 (0.185)	0.2881	NS	1.25 (0.83-1.86)
block 2	CC	28.25 (0.130)	39.51 (0.096)	0.2014	NS	1.40 (0.84-2.33)
	CT	14.75 (0.068)	25.49 (0.062)	0.7894	NS	1.10 (0.56-2.13)
	GC	9.75 (0.045)	23.49 (0.057)	0.5028	NS	0.77 (0.36-1.66)
	GT	165.25 (0.758)	321.51 (0.784)	0.4552	NS	0.86 (0.58-1.27)

95% CI=95% confidence interval; NS=not significant.

## Discussion

Adrenomedullin (AM), a 52-amino acid smooth muscle-relaxing polypeptide, is produced in several tissues, including the eye [16]. In human eyes, AM has been identified in the aqueous humor that is produced by the iris ciliary body [17]. Studies in several mammalian species, including humans, have identified the pupillary sphincter as a target of ocular AM [18], and AM receptors linked to cAMP production have been identified [19]. The AM receptor is a complex molecule that consists of CALCRL and receptor activity–modifying protein 2 (RAMP2). CALCRL is a G protein-coupled receptor with seven transmembrane domains. The mechanism for its activation is unique; CALCRL is transported from endoplasmic reticulum to the cell membrane by RAMP2 where it is core glycosylated to become a receptor for AM [20]. The gene encoding CALCRL contains 15 exons interrupted by 14 introns, including one that spans more than 60 kilobases. Exons 1–3 constitute the noncoding region; exons 4 through 15 are coding elements, of which exons 8 to 14 encode seven transmembrane domains [21].

Previous animal studies have demonstrated that the AM gene is associated with abnormalities of vascular function or hypertension [22,23]. However mice that overexpressed *CALCRL* in smooth muscle-containing tissues had increased CALCRL/RAMP2 (AM receptors) in the pupillary sphincter muscle, resulting in enhanced AM-induced sphincter muscle relaxation. Importantly, certain transgenic mice had acutely and transiently elevated IOP between 1 and 3 months of age before chronically elevated IOP became evident. This indicates that overexpression of *CALCRL* can lead to increased sensitivity of the sphincter muscle to endogenous AM, thus causing chronic relaxation of the sphincter muscle and, as a consequence, obstruction of the aqueous outflow system.

In humans, PACG is characterized by reduced anterior chamber depth and increased lens thickness. In structurally predisposed eyes, drug-enhanced dilation of the pupil in dim light can provoke PACG [24,25]. Interestingly, dark-adapted transgenic mice exhibited impaired pupillary constriction in response to light exposure. However, topical administration of the AM antagonist AM-(20–50) normalized the pupillary response to light stimulation in the mice, suggesting an AM-mediated functional defect of the sphincter muscle. Taken together we expected that the degree of expression of *CALCRL* and the receptor activity of its product would affect the occurrence or development of angle closure glaucoma.

To our knowledge, this has been the first study to date investigating relationships between SNPs in *CALCRL* and PACG in a southern Chinese population. The results show rs1157699 is nominally associated with APACG with a marginal significance (uncorrected p value 0.024 and 0.028 observed in allele and genotype analyses, respectively, of this site). Moreover, our study suggests that the haplotype T_rs840617_C_rs6759535_T_rs1157699_ is positively associated with APACG and that the haplotype T_rs840617_C_rs6759535_C_rs1157699_ might be a protective haplotype for APACG. However we did not find any evidence of association between common variants of *CALCRL* and CPACG.

In single-marker analysis, we observed that the T_rs1157699_ allele is significantly more common in APACG patients than controls (uncorrected p=0.02), indicating a possible protective role of the C_rs1157699_ allele. Although rs1157699, which locates in the first intron of *CALCRL*, is not a functional SNP, it could have other effects, such as influencing splicing or regulatory processes by affecting the binding of transcription factors to the gene. Furthermore, as a tag SNP, the polymorphism is representative of many other variants, which could be regulatory in the function of the receptor. Also, it may be in linkage disequilibrium with functional variants.

With regard to the haplotype analysis, we found two haplotypes T_rs840617_C_rs6759535_T_rs1157699_ and T_rs840617_C_rs6759535_C_rs1157699_ that are associated with APACG. The former was found to be significantly higher in APACG patients, while the latter was found to be lower in APACG patients. The haplotype association with APACG could be a result of the presence of the T_rs1157699_ allele, providing significance to the implicated block. This explanation is supported by the fact that the allele frequencies of the other two tag SNPs constructing this haplotype are similar in the investigated populations. Furthermore, the haplotype association is statistically significant after the Bonferroni correction.

To our knowledge, this is the first molecular epidemiology study suggesting a possible association between *CALCRL* polymorphisms and APACG risk. Despite the significance of SNP rs1157699 not withstanding correction for multiple testing, the results of our exploratory analysis warrant further studies.

The lack of association between the tag SNPs and CPACG suggests that *CALCRL* is not a significant risk factor for its development. This may be because the mechanism causing CPACG is much more complicated than APACG [26]. In APACG attack, sphincter muscle palsy occurs and the pupil dilates, resulting in pupillary block. With CPACG, multiple mechanisms are involved, for example, closer location of iris root insertion to the angle and thicker and fleshier peripheral irises and anteriorly positioned ciliary bodies. Based on the above reasons, CPACG might be genetically different from APACG.

In conclusion, this study shows a possible association between *CALCRL* and APACG, and *CALCRL* may not play a major role in the etiology of CPACG. Additional replication studies are necessary to confirm such association.
